# Factors associated with quality of life of postmenopausal women living in Iran

**DOI:** 10.1186/s12905-020-00960-4

**Published:** 2020-05-14

**Authors:** Soheila Nazarpour, Masoumeh Simbar, Fahimeh Ramezani Tehrani, Hamid Alavi Majd

**Affiliations:** 1 Department of Midwifery, Chalous Branch, Islamic Azad University, Chalous, Iran; 2 Midwifery and Reproductive Health Research Center, Department of Midwifery and Reproductive Health, School of Nursing and Midwifery, Shahid Beheshti University of Medical Sciences, Vali-Asr Avenue, Cross of Vali-Asr and Hashemi Highway, Opposite to Rajaee Heart Hospital, Tehran, 1996835119 Iran; 3 Reproductive Endocrinology Research Center, Research Institute for Endocrine Sciences, Shahid Beheshti University of Medical Sciences, Tehran, Iran; 4 Department of Biostatistics, School of Paramedicine, Shahid Beheshti University of Medical Sciences, Tehran, Iran

**Keywords:** Menopause, Quality of life, Effective factors

## Abstract

**Background:**

Quality of life (QoL) after menopause could be influenced by a host of personal and social factors. This study aimed to determine the factors associated with quality of life among postmenopausal women.

**Methods:**

This cross-sectional study was conducted among 405 postmenopausal women selected using a multi-stage randomized sampling. The data-collection tools were the WHO Quality of Life-BREF (WHOQOL-BREF), the Menopause Rating Scale (MRS), and a researcher-designed questionnaire. The relationship between QoL and its potentially correlated factors was examined using *t*-test, ANOVA, Pearson’s correlation, Spearman’s correlation coefficient, and multiple linear regression.

**Results:**

A negative correlation was found between the scores of QoL (total and all subscales) and the MRS total scores. The total scores of QoL were negatively correlated with duration of menopause (*r* = − 0.127, *P* = 0.010), gravida (*r* = − 0.177, *P* < 0.001), parity (*r* = − 0.165, *P* = 0.001), frequency of stillbirth (*r* = − 0.104, *P* = 0.037), vaginal delivery (*r* = − 0.161, *P* = 0.001), and waist-to-hip ratio (*r* = − 0.195, *P* < 0.001). The QoL total scores were positively correlated with the educational level of the participants (*r* = 0.207, *P* < 0.001) and that of their spouses (*r* = 0.160, *P* = 0.001) along with their level of monthly family income (*r* = 0.218, *P* < 0.001). Multiple-linear-regression analysis showed that the total score of QoL decreased with inadequate income, waist-to-hip ratio, and the total score of MRS.

**Conclusions:**

Personal and social factors along with the severity of menopausal symptoms affect QoL post-menopause. These factors need to have a bearing on any effort to improve QoL among postmenopausal women.

## Background

The World Health Organization defines quality of life (QoL) as “individuals’ perception of their position in life in the context of the culture and value systems in which they live and in relation to their goals, expectations, standards and concerns [1, 2].”

Quality of life is a wide-ranging phenomenon that is affected in a complex way by an individual’s physical health, psychological state, level of independence, social relationships, and personal beliefs, along with their relationship to the salient features of their environment [3, 4].

QoL is also an aspect of health that plays a significant role in the conduct and evaluation of health interventions [5]. Thus research on QoL helps pave the way for more effective treatments and rehabilitative programs [6].

New developments in the medical sciences suggest that life expectancy has increased globally [7]. Today, many women spend a third of their lives after menopause [8]. Therefore, the QoL of postmenopausal women is of great public-health interest [9].

Menopause is an episode in women’s lives that has physical, psychological, and social consequences, and thereby affects QoL. Symptoms experienced during menopause and socio-demographic characteristics affect OoL in postmenopausal women [10, 11].

The primary effects of menopause are associated with estrogen deficiency. The main health concerns of postmenopausal women include vasomotor symptoms, urogenital atrophy, osteoporosis, cardiovascular disease, cancer, decreased cognitive function, and sexual problems [12].

Hormonal changes that begin during the menopausal transition result in physiological changes and various other symptoms. For this reason, women often experience a wide range of symptoms, including hot flashes, insomnia, weight gain and bloating, mood swings, irregular menstruation, breast pain, depression, and headaches. These symptoms could be distressing, particularly as they occur at a time when women have important roles in society, within the family and at the workplace [13].

The menopause-related conditions lead to reduced quality of life among women [13, 14].

Several studies have revealed a set of factors that could be associated with QoL [11, 15]. Contradictions could nevertheless be found in different studies. The study aimed to find out the factors that are associated with quality of life of postmenopausal women.

## Methods

### Study design

This is a cross-sectional study of 405 postmenopausal women, aged 40 to 65 years, residing in the cities of Chalous and Noshahr, two cities located in northern Iran. This study began in October 2013 and ended in May 2014. This study is part of an extensive study of a PhD thesis.

The population of present study was the participants of a previous study conducted to evaluate the sexual function of postmenopausal women [16]. In these women, natural menopause had occurred over the past 3 years and they had no history of serious physical or mental illness. A stratified, multi-stage probability-cluster sampling was carried out through the probability-proportional-to-size (PPS) procedure.

Out of 620 postmenopausal women that were checked for inclusion criteria, 505 women (81.5%), met our inclusion criteria, out of them 420 women agreed to participate in the study and signed the written informed consent; finally, 405 women returned the complete questionnaire.

The calculation of sample size was based on the population proportion = 0.60, α = 0.05, ε = 0.1 and the design effect of 1.5.

The eligible women were explained about the study and invited to participate in the study. Those who agreed to participate completed the informed consent form.

The primary response rate of the postmenopausal women who were contacted, the eligibility rate among those who agreed to be participated, and the rate of participation among those found to be eligible were 82%, 96%, and 98%, respectively. The reason for not participating in the study may be due to menopausal complications.

### Measures

A detailed researcher-designed questionnaire comprising questions about personal and social characteristics, the World Health Organization Quality of Life-BREF (WHOQOL-BREF) [17, 18], and the Menopause Rating Scale (MRS) [19–21] were completed by all of the participants over the course of face-to-face interviews. The questionnaires were anonymous and were filled out by interviewers who were trained by the researchers and had sufficient information about the subject, enabling them to explain ambiguous cases to the participants.

The WHOQOL-BREF questionnaire, which measures overall QoL and particular aspects of it, has four domains of physical health, psychological health, social relations, and environment. The questionnaire contains 24 questions to assess these aspects of QoL, and two more questions about an individual’s overall perception of their QoL and health. After performing the calculations, a score ranging from 4 to 20 is obtained for each domain, and a score ranging from 16 to 80 is obtained as a total score, with higher scores indicating better QoL*.* These scores are in turn convertible to score points in the range of 0 to 100 [17, 18].

This tool has been developed in more than 15 countries and translated into multiple languages, with the questions’ concepts remaining the same in all of its versions [22]. In Iran, the standardization of the questionnaire was conducted by Nejat et al. [23], the results of which demonstrated the reliability and validity of the tool among healthy and patient groups. In Soltani et al.’s study also Cronbach’s alpha was calculated as 0.84 for this questionnaire [24] In present study its reliability was assessed using the test-retest method, resulting in a Cronbach’s alpha and intraclass correlation coefficient (ICC) of 0.921 and 0.968, respectively.

The Menopause Rating Scale (MRS) is a standard scale for rating the severity of menopausal symptoms in the three categories of somatic (vasomotor), psychological, and urogenital symptoms. The questionnaire consists of 11 questions, the responses to which are scored on a 5-score scale for the symptoms’ severity, including “none” (0), “mild” (1), “moderate” (2), “severe” (3) and “very severe” (4). A total score ≥ 17 is taken as indicating severe menopausal symptoms [19]. The validity and reliability of the Persian version of the questionnaire was confirmed by Masjoudi et al. In this study Internal consistency was 0.83 based on Cronbach’s alpha indicating the high reliability of the scale [25]. For the purposes of our study, the validity of the MRS was confirmed using content and face validity, and its reliability was assessed using the test-retest method, yielding a Cronbach’s alpha and intraclass correlation coefficient (ICC) of 0.933 and 0.977, respectively.

The researcher-designed questionnaire consisted of 28 questions about anthropometric, socio-demographic and reproductive characteristics. The heights and weights of the women were measured using the calibrated Balance Beam Doctor/Physician Scale with Height Rod (Detecto 439, 400 lbs., Made in the USA) (Amazon 2017), and then were used to calculate body mass index (BMI) (kg/m2).

### Statistical analysis

The relationship between QoL and its potentially correlated factors was assessed using *t*-test, ANOVA, Pearson’s correlation (for the normal-distribution quantitative variables), Spearman’s correlation coefficient (for the categorical variables), and multiple linear regression. The data were analyzed using the statistics software SPSS (version 18). The level of significance was set at *P* less than 0.05.

For the multiple-linear-regression models, we assumed that the personal and social factors along with the severity of menopausal symptoms were related to scores of QoL (total score and the scores of its domains). In these analyses, total QoL score and the scores of its domains (in separate calculations) were taken as the dependent variable. The independent variables in our model, which were entered as indicators, included the total score of MRS, age, duration of menopause, gravida, parity, frequency of vaginal delivery, frequency of stillbirth, waist-to-hip ratio, BMI, occupation, husband’s job, educational level of the women and that of their spouses, and level of monthly family income. These variables have been shown by other studies to be related to QoL [11, 26–37]. All of these variables except for age and frequency of vaginal delivery remained in the model. In our regression analyses, R2 equaled 0.271, which showed that 27.1% of our outcome variable (total score of QoL) was explained by the variables included in the regression model. The interactions between the independent variables were also assessed, even though they were not included in the final model as they were not statistically significant.

## Results

Five hundred and five postmenopausal women participated in the study. The average age of the participants was 52.8 ± 3.7 (mean ± SD) years. The demographic and anthropometric characteristics of the participants were as follows: 51.2 ± 3.5 years for age at menopause, 19.8 ± 14.4 months for duration of menopause, 29.5 ± 5.5 for BMI (Kg/m2), 0.9 ± 0.1 for waist-to-hip ratio, 4.7 ± 2.3 for number of pregnancies, and 4.1 ± 2.0 for number of deliveries. 80.0% of the participants were housewives and the educational level of most of them was low (63.5% were illiterate or merely knew reading and writing). Monthly household income was less than adequate in 65.4% of the women (Table 1).

**Table 1:** Distribution of anthropometric, socio-economic and reproductive characteristics (*N* = 405)

Variables	Mean ± SD/N (%)
Age (years)	52.8 ± 3.7
Age at menopause^a^ (years)	51.2 ± 3.5
Duration of menopause (months)	19.8 ± 14.4
Duration of marriage (years)	32.6 ± 6.4
BMI (Kg/m 2)	29.5 ± 5.5
Waist to Hip Ratio	0.9 ± 0.1
Gravida^b^	4.7 ± 2.3
Parity^c^	4.1 ± 2.0
Occupation	
Housewife	324 (80.0)
Employed	81 (20.0)
Husband’s job	
Employed	303 (75.0)
Retired or unemployed	101 (25.0)
Education	
Illiterate or merely reading and writing	257 (63.5)
Diploma and under diploma	112 (27.7)
Higher diploma	36 (8.9)
The adequacy of Monthly household income	
Less than adequate	265 (65.4)
Adequately or More	136 (33.6)

^a^ Menopause is defined as the time when there has been no menstrual periods for 12 consecutive months; ^b^ Number of pregnancies; ^c^ Number of deliveries

The total score of QoL was 54.53 ± 7.18 (in the range of 30.0 to 71.0). The highest and lowest scores belonged to the physical-health domain (14.28 ± 2.41 in the range of 7.0 to 19.0) and the psychological-health domain (12.75 ± 2.07 in the range of 7.0 to 18.0), respectively.

Our analysis of the MRS questionnaires yielded a mean ± SD total MRS score of 12.45 ± 7.20. The psychological and urogenital domains accounted for the highest and lowest scores, respectively (4.90 ± 3.45 and 3.10 ± 2.46, respectively). Overall, 29.1% of the participants reported severe menopausal symptoms (≥17).

The total scores of QoL were found to be in a significant negative correlation with the total scores of MRS and the scores in all of its domains (somatic, psychological and urogenital) (*P* < 0.001). All QoL domain scores were also negatively correlated with the total scores of MRS. Saving the environment domain of QoL – which did not show a significant correlation with the somatic domain of MRS – there was a significant negative correlation between the scores in the QOL domains (physical health, psychological health, social relations) and the scores in the MRS domains (Table 2).

**Table 2:** Quality of life (WHOQOL-BREF) and Menopause Rating Scale (MRS) (*N* = 405)

The severity of menopausal symptoms	Quality Of life				
	Physical health	Psychological health	Social relations	Environment	Total score
	*r*	*r*	*r*	*r*	*r*
Somatic	−0.399^***^	− 0.167^**^	− 0.188^***^	−0.125	−0.269^***^
Psychological	− 0.421^***^	−0.383^***^	− 0.251^***^	−0.183^***^	−0.390^***^
Urogenital	−0.266^***^	− 0.196^***^	−0.319^***^	− 0.147^**^	−0.299^***^
Total score	− 0.449^***^	−0.316^***^	− 0.303^***^	−0.168^**^	−0.394^***^

*P < 0.05. **P < 0.01. ***P < 0.001. Test: Pearson correlation coefficient

The duration of menopause was found to be reversely correlated with the total score of QoL (*P* = 0.010, *r* = − 0.127) and the scores of the psychological-health and environment domains (*P* < 0.05). The age of the women and their spouses, menopausal age, and duration of marriage were not found to be significantly correlated with the QoL scores (Table 3).

**Table 3:** Unadjusted correlation between QoL with demographic and reproductive characteristics (*N* = 405)

Characteristics	Quality of life				
	Physical health	Psychological health	Social relations	Environment	Total score
	*r*	*r*	*r*	*r*	*r*
Test: Pearson correlation coefficient					
Age (years)	− 0.054	− 0.090	− 0.052	− 0.067	−0.081
Husband’s age (years)	−0.056	− 0.028	− 0.054	− 0.083	− 0.069
Age at menopause (years)	−0.046	− 0.058	− 0.042	− 0.037	−0.057
Duration of menopause (months)	− 0.076	−0.140**	− 0.089	−0.109*	−0.127*
Duration of marriage (years)	−0.068	− 0.078	− 0.007	− 0.034	−0.057
BMI (Kg/m 2)	− 0.078	−0.029	− 0.067	−0.088	−0.013
Waist to Hip Ratio	−0.160**	− 0.210***	−0.112*	− 0.151**	−0.195***
Test: Spearman’s correlation coefficient					
Education level of the women	0.166**	0.254***	0.084	0.266***	0.207***
Education level of the husbands	0.120*	0.208***	0.049	0.199***	0.160**
Gravida	−0.182***	− 0.158**	−0.119*	−0.173***	−0.177***
Parity	− 0.183***	−0.157**	− 0.103*	−0.164**	−0.165**
frequency of stillbirth	−0.089	− 0.055	−0.070	− 0.129**	−0.104*
Vaginal delivery	− 0.181***	−0.136**	− 0.108*	−0.152**	−0.161**

*P < 0.05; **P < 0.01; ***P < 0.001

The education level of the participants (*P* < 0.001, *r* = 0.207) and their husbands (*P* = 0.001, *r* = 0.160) showed a significant positive correlation with the total scores of QoL (Table 3).

The total scores of QoL (*P* < 0.001) and the scores in most of its domains were significantly lower in the group with a less-than-adequate family monthly income, comparing to the group with sufficient income levels (Table 4).

**Table 4:** Quality of life based on socio-economic factors

Quality Of life	The adequacy of Monthly household income		*t*-Test (*P*)
	Less than adequate *N* = 265	Adequately or More *N* = 136	
Physical health	14.14	14.55	0.106
Psychological health	12.35	13.54	< 0.001
Social relations	13.89	14.54	0.016
Environment	12.99	14.19	< 0.001
Total score	53.37	56.82	< 0.001
Quality Of life	Occupation		*t*-Test (*P*)
	Housewife *N* = 324	Employed *N* = 81	
Physical health	14.07	15.11	< 0.001
Psychological health	12.62	13.28	0.009
Social relations	14.09	14.16	0.838
Environment	13.34	13.59	0.299
Total score	54.12	56.15	0.023
Quality Of life	Husband’s job		*t*-Test (*P*)
	Employed *N* = 303	Retired or unemployed *N* = 101	
Physical health	14.19	14.51	0.238
Psychological health	12.66	13.05	0.098
Social relations	14.07	14.25	0.535
Environment	13.22	13.55	0.053
Total score	54.13	55.70	0.157

Similarly, there was a positive correlation between family monthly income and the total scores of QoL (*P* < 0.001, *r* = 0.218), as well as the scores in all of its domains except social relations (*P* < 0.001).

The total score of QoL (*P* = 0.023), along with the scores in the physical-health and psychological-health domains (*P* < 0.001 and *P* = 0.009, respectively), was significantly lower among the housewives compared to the employed women (Table 4).

There was a significant reverse correlation between the scores of QoL (total scores and the scores in all of its domains) and the frequency of pregnancy (gravid) and delivery (*P* < 0.001, *r* = − 0.177 and *P* = 0.001, *r* = − 0.165, respectively, with the total QoL scores). Furthermore, the total scores of QoL and the scores in its environmental-health domain were reversely correlated with the frequency of stillbirth (*P* = 0.037, *r* = − 0.104 and *P* = 0.009, *r* = − 0.129, respectively) (Table 3).

The same type of correlation existed between the scores of QoL (total scores and all of the domain scores) and frequency of vaginal delivery (*P* = 0.001, *r* = − 0.161 with the total scores) (Table 3).

The scores of QoL were negatively correlated with waist-to-hip ratio (*P* < 0.001, *r* = − 0.195 with the total scores), but not significantly correlated with body mass index (BMI) (Table 3). It must be noted that the results mentioned above were yielded by unadjusted analyses.

Multiple linear regression showed that the total score of QoL decreased with inadequate income (*P* = 0.002, B = 2.318), waist-to-hip ratio (*P* = 0.004, B = -11.380), and the total score of MRS (*P* < 0.001, B = -0.420). The results of the multiple linear regression are shown in Table 5.

**Table 5:** Factors associated with quality of life among postmenopausal women (multiple linear regression) (*N* = 405)

Quality of life	Variables	Multiple linear regression		
		B	*P* value	
Physical health	Waist-to-hip ratio	−2.641	0.046	
	Total score of MRS	−0.154	< 0.001	
Psychological health	The adequacy of monthly household income^a^	0.816	< 0.001	
	Waist-to-hip ratio	−3.264	0.005	
	Total score of MRS	−0.098	< 0.001	
Social relations	The adequacy of monthly household income	0.610	0.032	
	Waist-to-hip ratio	−3.244	0.034	
	Total score of MRS	−0.115	< 0.001	
Environment	The adequacy of monthly household income	0.950	< 0.001	
	Total score of MRS	−0.053	< 0.001	
Total score	The adequacy of monthly household income	2.318	0.002	
	Waist-to-hip ratio	−11.380	0.004	
	Total score of MRS	−0.420	< 0.001	

^**a**^Classification of the adequacy of Monthly household income: 0 (Zero). Less than adequate; 1. Adequately or more

## Discussion

This study showed that out of various anthropometric, socio-economic and reproductive characteristics that may have influence the quality of life of postmenopausal women, waist-to-hip ratio, the severity of menopausal symptoms, and the adequacy of monthly household income are the most remarkable ones.

The results showed that QoL is negatively associated with the severity of menopausal symptoms. That is, for every one unit increase in the total score of MRS, the total score of QoL decreased by 0.420 units. This finding is in agreement with those of multiple studies showing the severity of menopausal symptoms to be in a negative association with QoL [29, 30, 32, 33, 38–40]. According to some estimates, 50 to 80% of women experience menopausal symptoms (physical or psychological), leading to a decline in QoL [15]. Menopause affects all aspects of health, and our study shows that QoL among postmenopausal women is jeopardized with the emergence of menopausal symptoms.

This study found family monthly income to be associated with QoL. The total score of QoL was 2.318 units higher among the women with adequate or higher monthly income comparing to those with less-than-adequate monthly income. The relationship between income/economic status and QoL has been confirmed in several studies [11, 26, 27, 34, 41, 42], all of which are consistent with our study. This finding might stem from the fact that women with higher socioeconomic statuses, due to proper nutrition and timely referrals to medical specialists, find it easier to cope with the side effects of menopause [27]. Moreover, favorable economic conditions are often associated with better-paying jobs and higher education levels in couples, which could in turn ease their adaptation to the changes during menopause and senility. What is well established is that QoL during menopause is influenced in consequential ways by physical, emotional, social, and economic factors [26].

No significant association was found between QoL and BMI, which is consistent with the findings of Fallahzade et al. [27] and Golmakany et al. [35], but inconsistent with those of several other studies [28, 31, 41, 43, 44]. In study of Fallahzadeh [28], women with a BMI equal to or less than 18.5 kg/m^2^ had better QoL.

The finding demonstrated waist-hip ratio, however, to be inversely associated with QoL, so that with each unit increase in waist-hip ratio the total score of QoL decreased by 11.380 units. Considering that waist-to-hip ratio is an indicator of obesity, QoL could be theoretically tied to obesity, but there remains a need for further investigation on this front.

The results also showed a negative correlation between duration of menopause and the total score of QoL, as well as its psychological-health and environment domains. This finding has been confirmed by other studies [36, 37]. Some studies, however, showed that women whose menopause was 5 years old or older showed better quality of life [28, 43]. While their study examined duration of menopause in two groups and with a five-year-old threshold, our study was performed with more precision and duration of menopause was calculated up to months. Therefore, this controversy could be attributed to the different method of data analysis and the study population.

There was not any significant correlation between age and QoL, which does not correspond with a number of studies [11, 27, 30, 37, 41, 43, 45, 46] and could be the result of differences in study population and data-collection tools.

A positive correlation was found between level of education (among the women and their spouses) and the total score of QoL along with all of its domain scores except the domain of relationships. This finding has also been confirmed elsewhere [11, 15, 27, 30, 36, 37, 41, 43, 46, 47].

Surely women with higher education levels have greater access to credible sources of information and health resources, and hence are more equipped to deal with the complications and symptoms of menopause, which in turn leads to better QoL [27]. On the other hand, higher education is often synonymous with higher income levels and more opportunities in one’s professional and social lives [11], which could also improve QoL.

The present study revealed that the total scores of QoL and the scores in the physical- and psychological-health domains were lower among the housewives compared with the employed women. The spouses’ occupations, however, showed no correlation with the QoL of the participating postmenopausal women. The relationship between employment status and QoL has been examined by several studies [27, 36, 41, 43, 46, 48–50], most of which found employed women to have a better QoL than housewives [27, 36, 43, 48], which is consistent with our study.

Norozi et al. have showed that retired postmenopausal women enjoy a better QoL than housewives [46], while the study of Shobeiri et al. found that QoL is stronger among housewife postmenopausal women comparing to employed women [41].

It could be speculated that a job or responsibility in an organization boosts confidence among middle-aged women and helps to improve their QoL [46]. The employed women seemed that had better social support [41]. Housewives, on the other hand, are less exposed to the social environment outsides their homes and are mostly occupied with household chores, personally carrying out many of the tasks that working, financially independent women often delegate to others.

The side effects of menopause could be associated with the burdens of housework, which further complicate matters and diminish QoL. Working women also tend to have better mental health than stay-at-home ones [27], and given their routine exposure to social settings outside the home, are better able to withstand the symptoms of menopause.

This study showed that QoL is in a negative correlation with the frequencies of pregnancy (gravida), delivery (parity), and vaginal birth. Consistent with our findings, the Fallahzadeh et al. study [27] showed that the frequency of pregnancy could be correlated with QoL, and Monterrosa-Castro [31] showed that QoL significantly decreases with parity. Some studies also showed that number of children was inversely associated QoL in postmenopausal women [41, 51]. Given the association of the number of children with the frequencies of pregnancies and delivery, this result is consistent with the result of the present study.

These findings indicate that multiparity and multiple childbirths could cause physical and psychological impairments and hence have a negative impact on QoL. Higher number children could increase parental stress and responsibility as well as financial problems [52]. Mothers’ concerns regarding childcare – providing a decent life for them, paying attention to comfort them - always take precedence over their own convenience, thus correlating with their QoL. It could also be inferred that the odds of stillbirth are increased with every pregnancy, as a significant negative correlation was found between QoL and the frequency of stillbirth.

### Strengths and limitations

To mention the strengths of the present study, we excluded women whose menopause was longer than 3 years at the time of the study. It could have weakened the effects of aging on QoL. In addition, our study was conducted among general, Iranian postmenopausal women, which decreased participation bias.

Regarding limitations, the present study was conducted in two cities located in northern Iran, and its results may not be generalizable to other communities with different attitudes, customs, cultures, and lifestyles. Another important limitation of this study was that the present study was cross-sectional study and this design did not permit the assessment of the temporal sequence of QoL and effective factors, making it impossible to assess causal relations.

## Conclusion

Menopause and its symptoms tend to impact on quality of life, a metric that is under the influence of multiple personal and social factors among postmenopausal women. Some factors have a negative effect on quality. These factors include the severity of menopausal symptoms, duration of menopause, gravida, parity, frequency of stillbirth, vaginal delivery, and waist-to-hip ratio. Other factors have a positive effect on the quality of life, such as the educational level of the postmenopausal women and that of their spouses, and level of monthly family income. Thus, appropriate interventions need to be made by way of public-health policies that aim to mitigate menopausal symptoms and maintain quality of life among these women. In this regard, these effective factors should be considered for planning of programs to improve the quality of life among postmenopausal women.

## Data Availability

The datasets used and/or analysed during the current study are available from the corresponding author on reasonable request.

## Abbreviations

QoL: Quality of life
WHO: World health organization
WHOQOL-BREF: World health organization quality of life-BREF
MRS: Menopause rating scale
ANOVA: Analysis of variance
ICC: Intraclass correlation coefficient
BMI: Body mass index
